# Butyrophilin 3A/2A1-independent activation of human Vγ9Vδ2 γδ T cells by bacteria

**DOI:** 10.1093/pnasnexus/pgaf358

**Published:** 2025-11-13

**Authors:** Daniel Gombert, Jara Simeonov, Katharina Klein, Sophie Agaugué, Alexander Scheffold, Dieter Kabelitz, Christian Peters

**Affiliations:** Institute of Immunology, Christian-Albrechts-University and University Hospital Schleswig-Holstein Campus Kiel, Kiel 24105, Germany; Institute of Immunology, Christian-Albrechts-University and University Hospital Schleswig-Holstein Campus Kiel, Kiel 24105, Germany; Institute of Immunology, Christian-Albrechts-University and University Hospital Schleswig-Holstein Campus Kiel, Kiel 24105, Germany; ImCheck Therapeutics, Marseille 13009, France; Institute of Immunology, Christian-Albrechts-University and University Hospital Schleswig-Holstein Campus Kiel, Kiel 24105, Germany; Institute of Immunology, Christian-Albrechts-University and University Hospital Schleswig-Holstein Campus Kiel, Kiel 24105, Germany; Institute of Immunology, Christian-Albrechts-University and University Hospital Schleswig-Holstein Campus Kiel, Kiel 24105, Germany; Cyto Kiel - Flow Cytometry Core Facility, Christian-Albrechts-University and University Hospital Schleswig-Holstein Campus Kiel, Kiel 24105, Germany

**Keywords:** bacteria, butyrophilin, gamma delta T cells, interleukin-18, phosphoantigen

## Abstract

The activation of human Vδ2 γδ T cells by phosphoantigens (pAg) strictly depends on transmembrane butyrophilin (BTN) molecules, specifically BTN3A isoforms and BTN2A1. Several bacteria, including *M. tuberculosis*, produce potent pAg and thus trigger a strong activation of Vδ2 T cells. The antigen-specific activation of CD4 and regulatory (Treg) T cells can be monitored by the rapid upregulation of CD154 and CD137, respectively. We have previously established that CD137 is also rapidly upregulated on Vδ2 T cells upon stimulation with pAg. In the present study, we have used antagonistic anti-BTN3A/2A1 antibodies to dissect the pAg-dependent and pAg-independent activation of Vδ2 T cells by various microbes. While the activation of Vδ2 T cells by pAg and aminobisphosphonate zoledronate was completely blocked by anti-BTN3A/2A1 antibodies, only partial inhibition was observed for activation with *M. tuberculosis* and other bacteria as analyzed by CD137/CD154 upregulation and intracellular interferon-γ expression. Similarly, anti-TCR antibody 7A5 and Lck inhibitor emodin had only a minimal inhibitory effect on activation by bacteria but strongly reduced pAg activation of Vδ2 T cells. Further studies revealed a crucial role of IL-18 in the BTN/TCR-independent early activation of Vδ2 T cells by bacteria. Neutralizing anti-IL-18 antibodies and inflammasome inhibition did not affect pAg activation of Vδ2 T cells but strongly reduced their activation by bacteria. Our results identify a BTN/TCR-independent but IL-18 and inflammasome-dependent activation pathway of Vδ2 T cells, which might be relevant for the role of Vδ2 T cells during bacterial infections.

Significance Statement Human Vδ2 γδ T cells are selectively activated by phosphoantigens (pAg) derived from microbes or tumor cells. The activation of Vδ2 T cells by pAg is absolutely dependent on transmembrane butyrophilin (BTN) BTN3A and BTN2A1 molecules. In line, the activation of Vδ2 T cells by pAg is completely abrogated by inhibitory anti-BTN antibodies. Unexpectedly, however, such antibodies had only a minor inhibitory effect on the microbe activation of Vδ2 T cells. We also found that IL-18 and inflammasome activation play a major role in the pAg-independent activation of Vδ2 T cells. Our studies reveal novel insights into the microbial activation of Vδ2 T cells, which could reshape our understanding of the role of γδ T cells in bacterial infections.

## Introduction

γδ T cells constitute the third lineage of the adaptive immune system but share many features of innate immune cells. The most abundant γδ T-cell population in human peripheral blood, Vγ9Vδ2 T cells (termed Vδ2 in the following; 1–10% of CD3^+^ T cells), is selectively activated by pyrophosphate intermediates of the microbial nonmevalonate or the eukaryotic mevalonate pathway of isoprenoid biosynthesis. These “phosphoantigens” (pAg) differ in their potency, with microbial pAg such as (*E*)-4-hydroxy-3-methyl-but-2-enyl pyrophosphate (HMBPP) activating Vδ2 T cells at nanomolar concentrations, whereas endogenous intermediates of the mevalonate pathway such as isopentenyl pyrophosphate (IPP), require higher (micromolar) concentrations (1, 2). Such concentrations are not generated in healthy cells, but tumor cells often have a dysregulated mevalonate pathway and produce increased amounts of IPP, which are sufficient for Vδ2 T-cell activation (3). The mevalonate pathway can also be pharmacologically targeted, e.g. by inhibition of the farnesyl pyrophosphate synthase with the aminobisphosphonate Zoledronate (Zol), which induces intracellular accumulation of IPP in monocytes, leading to the activation of Vδ2 T cells (4, 5). Zol is commonly used to selectively activate and expand Vδ2 T cells (6).

The mechanism of pAg recognition by Vδ2 T cells has been elucidated in recent years (7). While neither classical nor nonclassical HLA molecules are involved, transmembrane butyrophilin (BTN) protein family members, specifically BTN3A1/2 and BTN2A1, are indispensable (8–10). In the presenting cells, pAg binds to the intracellular B30.2 domain of BTN3A1 and thereby enables the interaction with the intracellular domain of BTN2A1 (11). Following this interaction BTN2A1, which does not bind pAg, can interact with a lateral germline-encoded region between the CDR2 and CDR3 of the Vγ segment of the γδ TCR (10, 12). Upon intracellular BTN2A1/3A1 interaction, the extracellular IgV domain of BTN3A1/2 might directly interact with the γδ TCR CDR3 region, or alternatively, BTN3A1 might contribute to the recruitment and binding of a novel TCR ligand (11, 13). The BTN-dependent activation of Vδ2 T cells can be specifically modulated by agonistic or inhibitory antibodies. While Vδ2 T cells are selectively activated by agonistic anti-BTN3A antibodies (5, 8, 14), the pAg-stimulated activation is completely abrogated by inhibitory anti-BTN3A or anti-BTN2A1 antibodies (15, 16).

Looking at the early events of T-cell activation can help to identify rare antigen-specific T cells within a polyclonal T-cell population. Human conventional CD4 T cells (Tcon) rapidly upregulate CD154 (CD40-L) upon TCR-dependent antigen stimulation (17), whereas CD4 regulatory T cells (Treg) upregulate CD137 (4-1BB) more rapidly (18). However, at later time points, CD137 is also upregulated on CD4 Tcon in response to TCR-stimulation (19). T cells differentially expressing CD137 or CD154 can be isolated by magnetic or flow cytometry-based cell sorting for further functional and TCR repertoire analysis. This method has been successfully used to characterize the dynamics of CD4/Th17 T-cell and Treg responses to fungal antigens and allergens (20, 21) and more recently to characterize the CD4 T-cell response to SARS-Cov-2 (22).

We have previously established that human Vδ2 T cells rapidly upregulate CD137 upon Zol and pAg stimulation (23). While bacteria, including *M. tuberculosis*, strongly induce Vδ2 T-cell proliferation in vitro (24) and are known producers of pAg (25), here we identify an additional BTN3A/2A1-independent early activation of Vδ2 T cells by various bacteria and an important role of IL-18 in this process.

## Results

### Rapid upregulation of CD137 and CD154 upon activation of Vδ2 T cells

**Fig. 1.**
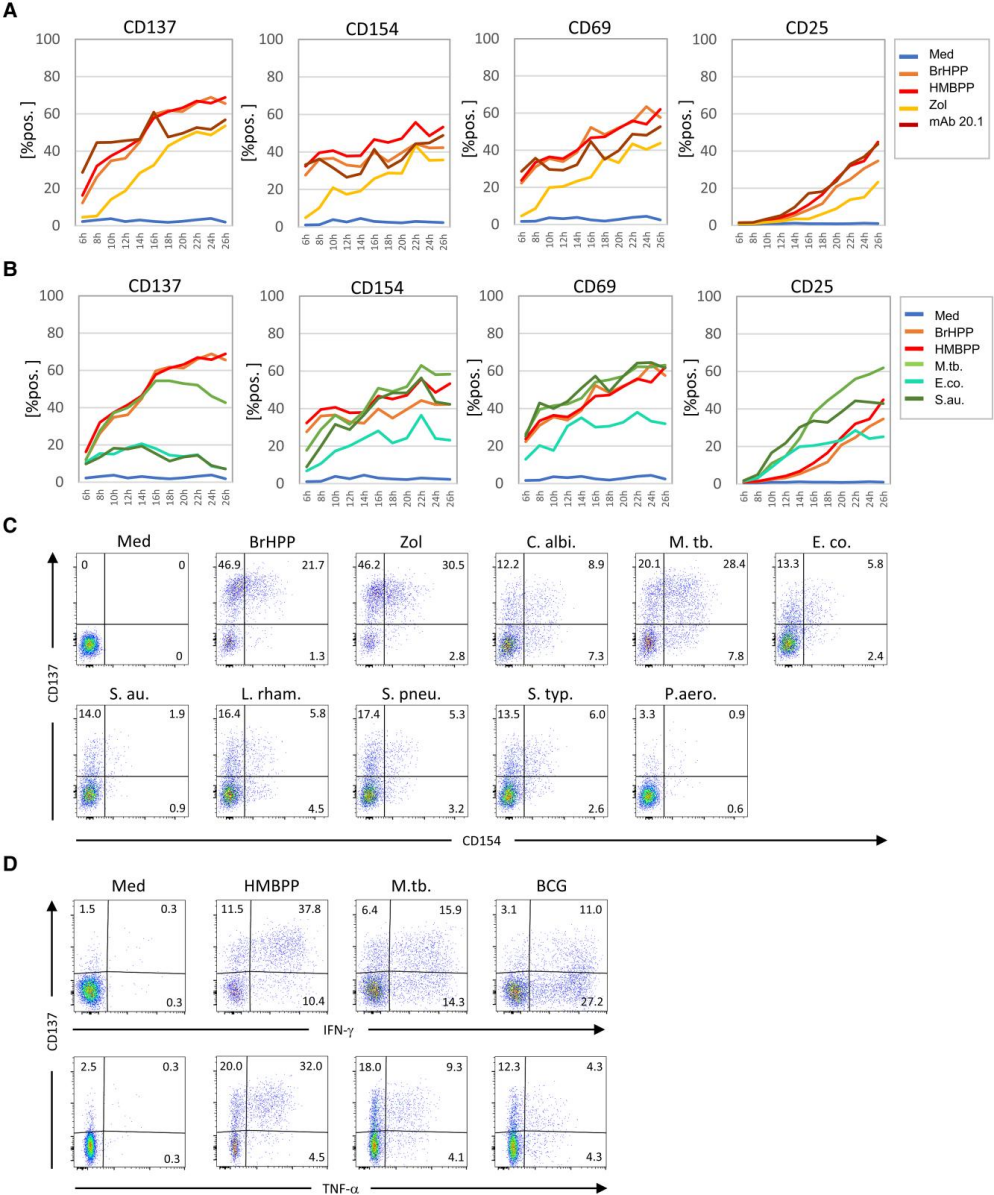
Kinetics of Vδ2 T-cell activation and microbe reactivity. PBMC from healthy donors were stimulated with A) different Vδ2 T-cell specific activating agents (BrHPP, HMBPP, Zoledronate [Zol] or agonistic anti-BTN3A mAb 20.1, or B and C) different heat killed bacteria (*M. tuberculosis* [M. tb.], *E. coli* [E. co.], *S. aureus* [S. au.], *L. rhamnosus* [L. rham.], *S. pneumoniae* [S. pneu.], *S. typhimurium* [S. typh.] and *Ps. aeruginosa* [P. aero.]) or fungi (*C. albicans* [C. albi.]), or D) viable Bacillus Calmette-Guérin [BCG]. A, B) After the indicated time points, PBMC were stained with anti-CD25, anti-CD69, anti-CD137, and anti-CD154 mAb surface expression, with a gate on CD3^+^Vδ2^+^ T cells was monitored by flow cytometry over a period of 26 h. The mean values of the proportion of positive cells within Vδ2 T cells of five independent experiments are shown. C) The association of CD137 and CD154 expression on Vδ2 T cells (gated as CD3^+^Vδ2^+^ cells within the PBMC) 16 h after initial stimulation is depicted in a dot plot from a representative experiment. D) Representative dot plot of the surface mobilization of CD137 and intracellular levels of IFN-γ and TNF-α 20 h after activation with indicated stimuli. For the detection of intracellular markers, monensin was added 4 h before fixation.

Upregulation of CD137 and CD154 is commonly used to detect antigen-specific Treg and Tcon αβ T cells, respectively (17, 18, 20–22). We have previously established that BTN3A-dependent activation of Vδ2 T cells by Zol and pAg can be monitored by upregulation of CD137 (23). Here, we extended this analysis by including various heat-killed and live bacteria and fungi to activate Vδ2 T cells within PBMC. We performed a time course kinetic analysis of the upregulation of CD137, CD154, CD69, and CD25 on Vδ2 T cells in response to pAg, Zol, and agonistic anti-BTN3A mAb 20.1 (Fig. 1A), and in response to microbes (Fig. 1B). Upon stimulation, CD69, CD137, and CD154 were rapidly upregulated on Vδ2 T cells (Fig. 1A). This upregulation occurred most rapidly in response to pAg BrHPP/HMBPP and mAb 20.1, was detectable already 6 h after activation, and reached a maximum after 20–24 h. CD25 upregulation was substantially delayed, with a clearly detectable induction observed only after 16 h. The upregulation by Zol was slower than by pAg/mAb 20.1, and became apparent after 10 h (CD69, CD137, CD154) or 18 h (CD25). Altogether, the clearest induction after selective activation of Vδ2 T cells was observed for CD137 (Fig. 1A). Next, we used a panel of different heat-killed microbes to monitor activation of Vδ2 T cells within PBMC. The stimulation with *M. tuberculosis* (originally described to induce strong Vγ9Vδ2 T cell activation and expansion) (24 ), also resulted in a clear upregulation of CD137 and CD154 (Fig. 1B, C). The kinetic of CD137 induction followed for 16 h in speed and magnitude the kinetic of pAg activation and leveled off thereafter. Also, the CD137 upregulation in response to *E. coli* and *S. aureus* followed a similar kinetic (with a peak after 14 h), but the overall magnitude of induction was much lower (Fig. 1B). The kinetic of CD69 and CD154 induction resembled for all three bacteria the activation by pAg. Interestingly, the upregulation of CD25 was substantially faster than by pAg activation and was already detectable after 10 h (Fig. 1B). Moreover, almost all other tested microbes led to CD137 and CD154 upregulation on Vδ2 T cells, although the magnitude of the response varied (Fig. 1C). Importantly, activation of Vδ2 T cells was not limited to killed bacteria (*M. tuberculosis*) but also occurred in response to live *Bacillus Calmette-Guérin* (BCG) which induced upregulation of CD137 and IFN-γ as well as limited TNF-α production (Fig. 1D). The upregulation of CD137 and CD154 on Vδ2 T cells by pAg did not strictly depend on the presence of accessory cells as shown with isolated γδ T cells. In contrast, Zol or microbe stimulation required the presence of accessory cell populations within the PBMC, reflecting the induction of IPP by Zol-activated monocytes or the phagocytosis of bacteria by monocytes (Fig. S1).

### BTN- and TCR-independent activation of Vδ2 T cells by bacteria

**Fig. 2.**
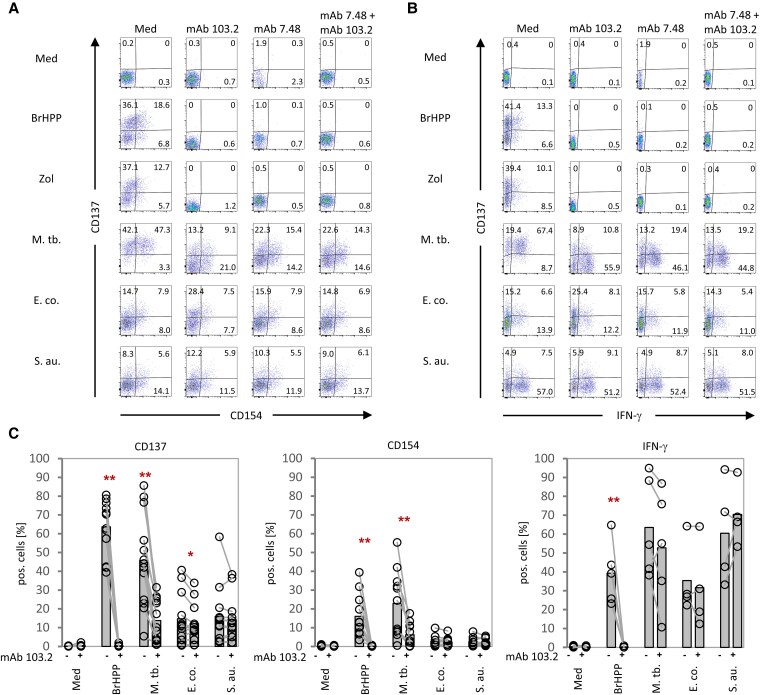
Vδ2 T-cell activation by different bacteria and the dependency on BTN molecules. PBMC from healthy donors were stimulated with different Vδ2 T-cell activating agents (Zoledronate [Zol], BrHPP), or different heat-killed bacteria (*M. tuberculosis* [M. tb.], *E. coli* [E. co.], and *S. aureus* [S. au.]. The surface mobilization of CD137 and CD154 on Vδ2 T cells was measured by flow cytometry after 16 h. For the intracellular detection of IFN-γ, monensin was added 4 h before fixation. A, B) The dot plots of a representative experiment show the effect of inhibitory anti-BTN3A (clone: 103.2) and anti-BTN2A1 (clone: 7.48) antibodies on CD137, CD154, and IFN-γ upregulation. C) The percentage of Vδ2 T cells positive for CD137 and CD154 and the effect of BTN3A blockade (mAb 103.2) is shown for different donors (*n* = 13 [CD137], *n* = 11 [CD154], *n* = 5 [IFN-γ]). Statistical significances are indicated by asterisks according to Student’s t-test (*P ≤ 0.05; **P ≤ 0.01).

We used inhibitory anti-BTN2A1 (mAb 7.48) and anti-BTN3A (mAb 103.2) antibodies to interrogate the role of BTN molecules in the activation of Vδ2 T cells. As illustrated in Fig. 2, the upregulation of CD137, CD154, and intracellular IFN-γ upon pAg (BrHPP) or Zol stimulation was completely abrogated by individual BTN2A1 or BTN3A blockade. In striking contrast, the upregulation of the same markers in response to bacteria stimulation was only partially or not at all inhibited by BTN antibodies, even in combination of BTN2A1 and BTN3A blockade (representative dot plots in Fig. 2A, B; summary of several experiments in Fig. 2C). Importantly, while the induction of the effector cytokine IFN-γ by pAg BrHPP or Zol was completely inhibited by BTN2A1/3A antibodies, such a blockade did not prevent the IFN-γ induction in response to *E. coli* or *S. aureus* and only slightly reduced the IFN-γ induction by *M. tuberculosis* (Fig. 2B, C). To ensure that this difference was not due to insufficient blockade of BTN molecules by the antibodies, we performed dose-titration experiments. The results revealed clear dose-dependent inhibition of HMBPP-induced CD137 upregulation and IFN-γ induction by BTN2A1/3A antibodies while the activation of Vδ2 T cells by *M. tuberculosis* was not significantly reduced even in the presence of 10 μg/ml of BTN2A1/3A antibodies (Fig. S2). These data clearly demonstrate that bacteria-induced activation of Vδ2 T cells is only partially dependent on BTN2A1/3A signaling.

**Fig. 3.**
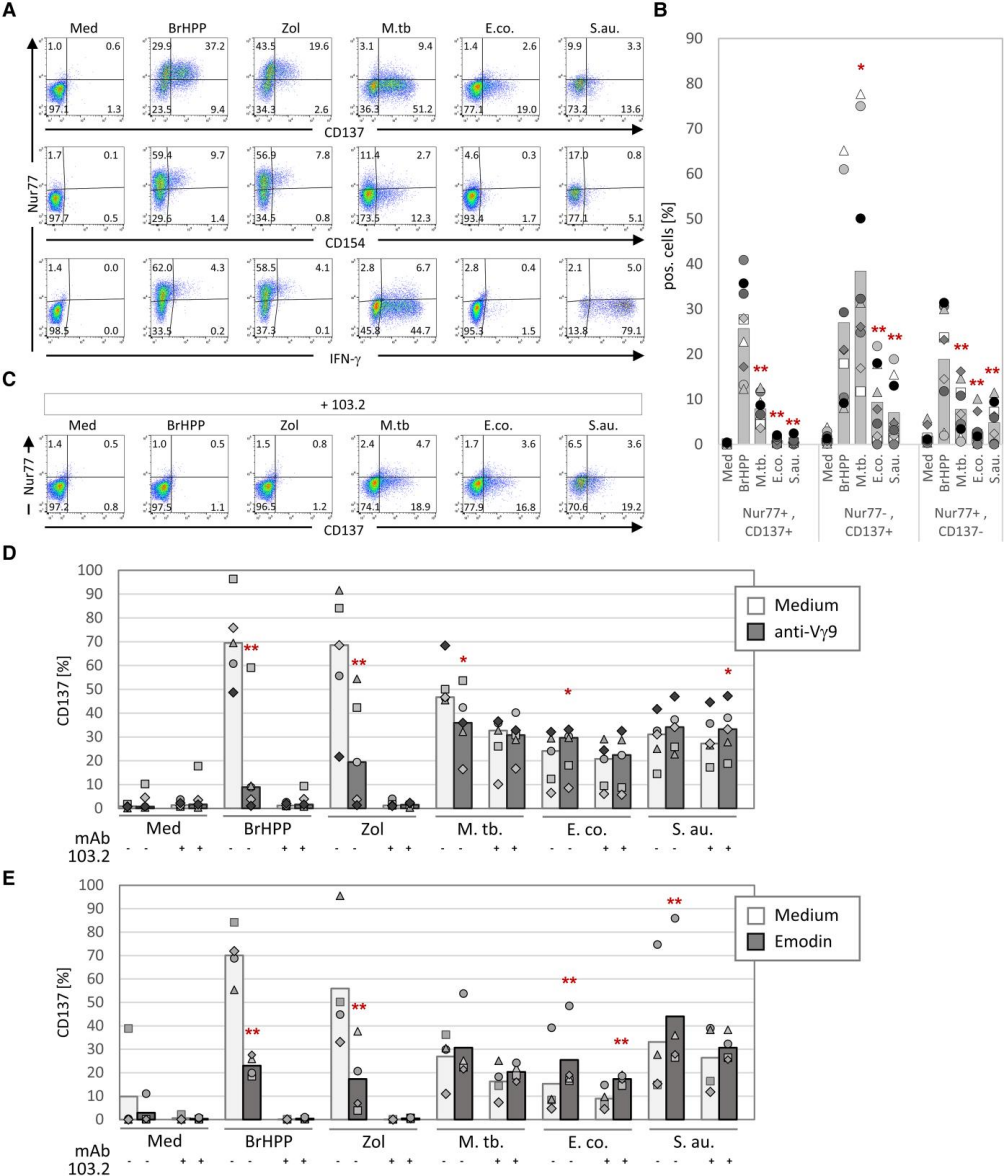
TCR dependency of bacterial Vδ2 T-cell activation. PBMC from healthy donors were stimulated with different Vδ2 T-cell activating agents (Zoledronate [Zol], BrHPP), or different heat-killed bacteria (*M. tuberculosis* [M. tb.], *E. coli* [E. co.], *S. aureus* [S. au.]. (A, B, C) CD137 and CD154 surface mobilization, IFN-γ production, and Nur77 induction in Vδ2 T cells were measured by flow cytometry 20 h after initial stimulation. A) The dot plots show the correlation of CD137, CD154, and IFN-γ with Nur77 in a representative experiment. B) The correlation of CD137 with Nur77 was quantified for different donors (*n* = 9). Asterisks indicate a significant difference from the respective BrHPP-treated sample according to Student's *t*-test (*P ≤ 0.05; **P ≤ 0.01). C) Using the same conditions as in (A), the BTN3A-dependent activation was blocked using mAb 103.2. D, E) The CD137 surface mobilization on Vδ2 T cells was quantified by flow cytometry 16 h after initial stimulation. To prevent the TCR-dependent activation D) anti-Vγ9 (clone: 7A5) Fab fragment (*n* = 5) or E) Emodin (40 µM) (*n* = 4) were added to the assay. BTN3A-dependent activation was blocked using mAb 103.2 as indicated. Asterisks indicate a significant difference from the respective untreated sample (medium) according to Student's *t*-test (*P ≤ 0.05; **P ≤ 0.01).

To investigate further the dependence on TCR-signaling of the bacteria-induced activation, we examined the induction of the transcription factor Nur77, which is a marker for antigen-specific TCR signaling (26). Nur77 was upregulated in Vδ2 T cells upon Zol and BrHPP stimulation, and Nur77 abundance correlated most strongly with CD137 and to a lesser degree with CD154 and IFN-γ (Fig. 3A, B). In contrast, only marginal Nur77-induction was observed in bacteria-activated Vδ2 T cells, and the induction of CD137, CD154, and IFN-γ was mainly found in Nur77-negative Vδ2 T cells (representative dot plots in Fig. 3A, summary of nine experiments in Fig. 3B). As expected, the induction of Nur77 in Zol and BrHPP-activated Vδ2 T cells was prevented by anti-BTN3A antibody 103.2 (Fig. 3C). In further support of a differential involvement of TCR signaling in pAg *versus* bacteria stimulation, we observed a strong inhibition of the BrHPP- and Zol-induced CD137 upregulation by the Fab-fragment of anti-Vγ9 mAb 7A5, which was again also completely inhibited by mAb 103.2 (Fig. 3D). Vγ9-blockade also reduced the *M. tuberculosis*-induced CD137 upregulation, but there was little or no effect of Vγ9-blockade upon *E. coli* and *S. aureus* activation (Fig. 3D). In the presence of Emodin, an inhibitor of tyrosine phosphorylation (mainly active on LCK) (27), the CD137 upregulation by BrHPP and Zol was significantly inhibited, whereas Emodin did not inhibit the CD137 upregulation in response to *M. tuberculosis*, *E. coli*, and *S. aureus* (Fig. 3E). Taken together, several independent lines of evidence indicate that the bacteria-induced activation of Vδ2 T cells is largely independent of TCR-signaling and LCK- or tyrosine-phosphorylation in general.

### Differential role of IL-18 in Vδ2 T-cell activation

**Fig. 4.**
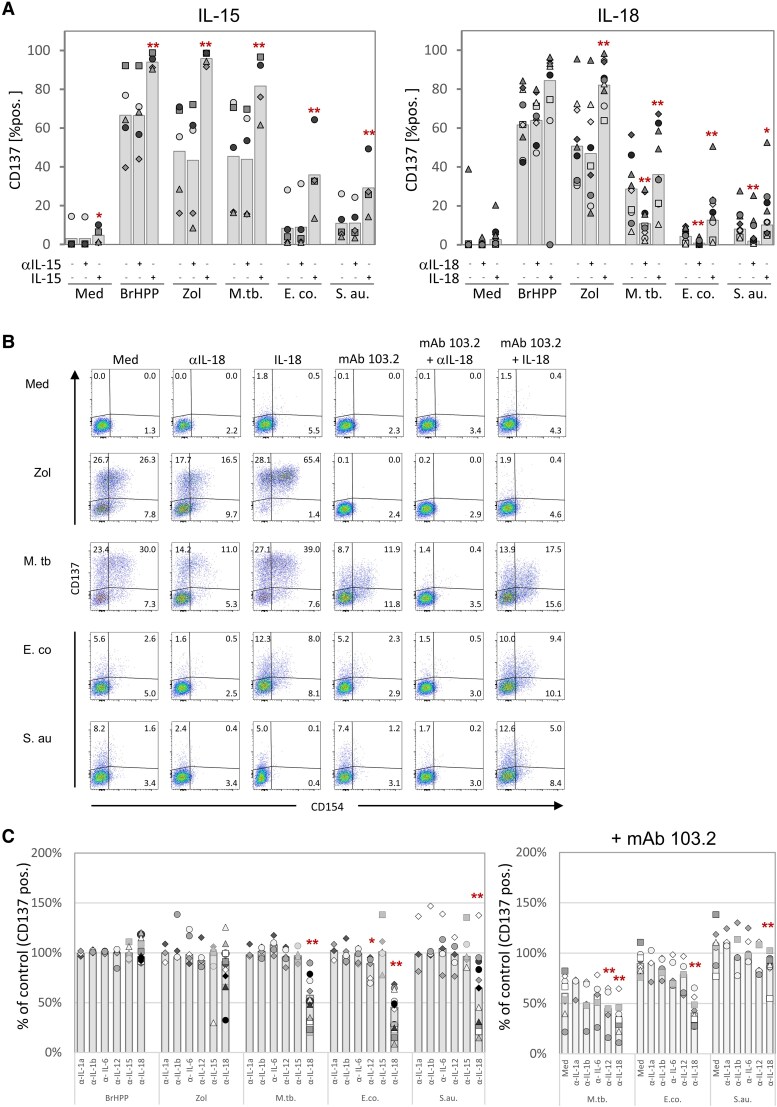
Influence of cytokines on Vδ2 T-cell activation. A) PBMC from healthy donors were stimulated or not [Med, Medium] for 16 h with BrHPP, Zoledronate [Zol], *M. tuberculosis* [M. tb.], *E. coli* [E. co.], and *S. aureus* [S. au.]. In addition, IL-15 (10 ng/mL), IL-18 (10 ng/mL), or neutralizing anti-IL-15 (10 μg/mL) or anti-IL-18 (0.1 µg/mL) antibodies were added as indicated. The proportion of CD137-positive cells among Vδ2 T cells was measured by flow cytometry. The results of five (IL-15 panel) or nine (IL-18 panel) experiments are shown. B) A representative dot plot illustrating the combined effects of IL-18, anti-IL-18 antibody, and BTN3A-blocking antibody 103.2. C) *Left:* PBMC were stimulated for 16 h as in (A) in the absence or presence of neutralizing antibodies against IL-1α (*n* = 3), IL-1β (*n* = 5), IL-6 (*n* = 5), IL-12 (*n* = 5), IL-15 (*n* = 5), or IL-18 (*n* = 17). *Right:* Additionally, the BTN3A-dependent activation was blocked by mAb 103.2 as indicated in the presence of anti-IL-1α (*n* = 3), anti-IL-1β (*n* = 5), anti-IL-6 (*n* = 5), anti-IL-12 (*n* = 5), anti-IL-15 (*n* = 5), or anti-IL-18 (*n* = 8)). The relative reduction of CD137 surface mobilization [%] compared to the corresponding untreated sample is presented in the histograms. Asterisks indicate a significant difference from the respective control according to Student's t-test (*P ≤ 0.05; **P ≤ 0.01).

As a next step we addressed the role of selected cytokines in the activation of Vδ2 T cells by pAg and bacteria since CD137 and CD154 expression has been described to be modulated by cytokines (28, 29). First, we measured various cytokines in the supernatants of PBMC activated with pAg, Zol, or bacteria in the presence or absence of mAb 103.2. Substantial amounts of IL-1α, IL-12p40, IL-15, and IL-18 were detected in response to bacteria, but very little, if any, upon BrHPP or Zol stimulation. Secretion of cytokines was not diminished in the presence of anti-BTN3A mAb 103.2 (Fig. S3). Based on the detected cytokine patterns and the known roles of IL-15 and IL-18 in promoting γδ T-cell activation (30–32), we then focused on IL-15 and IL-18. PBMC were again activated with BrHPP, Zol, or bacteria, and cell cultures were supplemented with exogenous IL-15 or IL-18, or neutralizing anti-IL-15 or anti-IL-18 antibodies to counteract possible effects of endogenous cytokines. As shown in Fig. 4A, both IL-15 and IL-18 significantly increased the CD137 upregulation on Vδ2 T cells in combination with all used stimuli. Interestingly, blocking of IL-18 (but not of IL-15) significantly decreased the CD137 upregulation induced by *M. tuberculosis*, *E. coli*, and *S. aureus* but did not affect CD137 upregulation triggered by BrHPP or Zol (in line with the failure of these stimuli to induce IL-15/IL-18; Fig. S3) (Fig. 4A). In view of the selective inhibitory effect of IL-18 blockade on CD137 upregulation upon bacteria stimulation, we next analyzed the reciprocal interplay between BTN3A and IL-18 by using combinations of neutralizing antibodies and exogenous IL-18. Dot plots of CD137 and CD154 induction of a representative experiment are shown in Fig. 4B. As before, upregulation of CD137/CD154 by Zol was completely blocked by mAb 103.2, even in the presence of exogenous IL-18. Upregulation by bacteria (most notably by *M. tuberculosis*) was partially inhibited by anti-IL-18 and anti-BTN3A mAb 103.2, and there was synergistic inhibition in the presence of both antibodies. Importantly, there was still strong upregulation of CD137 and CD154 in response to all three bacteria in the presence of mAb 103.2 provided that exogenous IL-18 was added. We extended these experiments to include neutralizing antibodies against IL-1α/β, IL-6, IL-12, and IL-15, in addition to anti-IL-18, again with or without additional BTN3A blockade by mAb 103.2 (Fig. 4C). Again, upregulation of CD137 on Vδ2 T cells in response to bacteria was reduced by anti-IL-18 alone but not by the other tested anti-cytokine antibodies (with the exception of a minor effect of anti-IL-12 on *E. coli* activation) (Fig. 4A, left). In the additional presence of mAb 103.2, inhibition of CD137 upregulation by anti-IL-18 was again more pronounced, not only in response to *M. tuberculosis* but also to *E. coli* and *S. aureus* (Fig. 4C, right). We conclude that IL-18 plays a major role in the BTN-independent part of Vδ2 T-cell activation by bacteria.

**Fig. 5.**
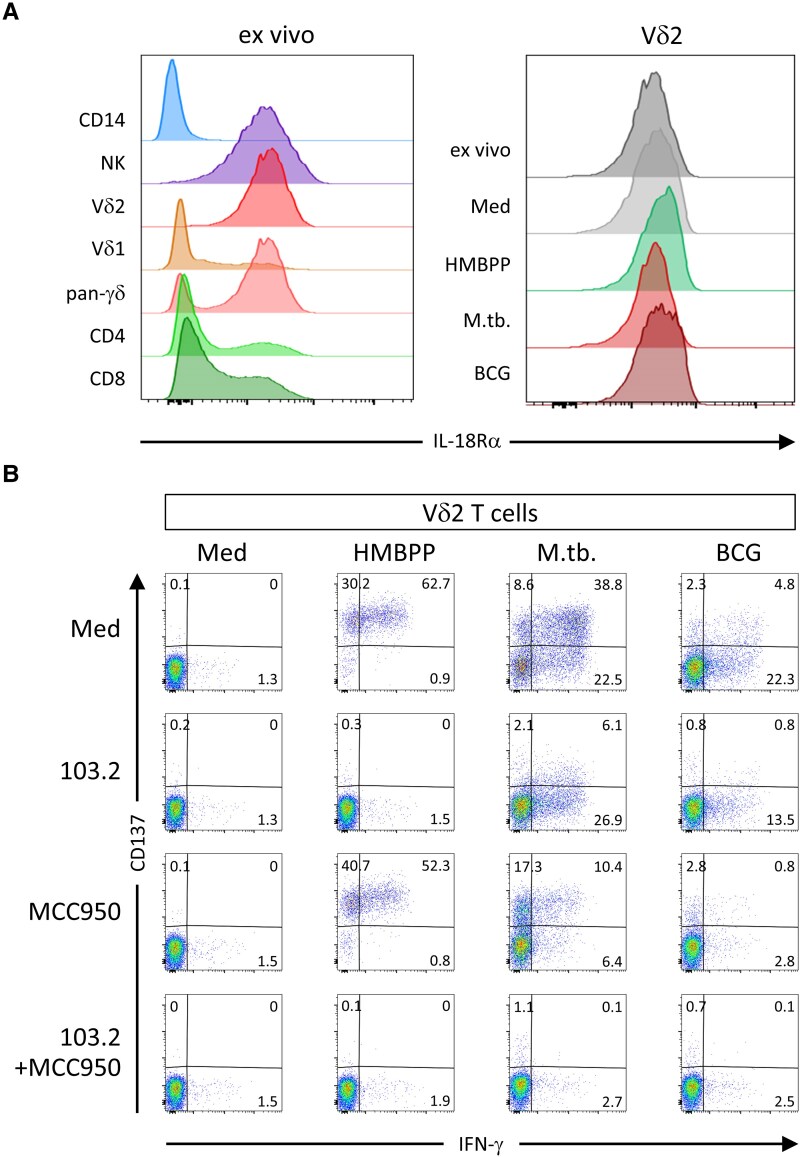
Impact of the inflammasome/IL-18 pathway on bacterial Vδ2 T-cell activation. PBMC from one representative healthy donor (out of at least four independent experiments) were stimulated for 20 h with HMBPP, *M. tuberculosis* [M. tb.], or Bacillus Calmette-Guérin [BCG], or remained unstimulated [Med, Medium]. A) The IL-18Rα (anti-CD218, clone: H44) surface abundance was measured by flow cytometry on different leukocyte populations (CD14+ monocytes, CD14-CD3-CD56+ NK cells, Vδ1, Vδ2, pan-γδ, CD4, and CD8 T cells) within freshly isolated PBMC (left panel) or on activated Vδ2 T cells within the PBMC (right panel). B) Effects of the BTN3A-blocking antibody 103.2 and MCC950 on the induction of CD137 and IFN-γ on Vδ2 T cells.

In view of the observed IL-18 reactivity, we next analyzed the expression of IL-18 receptor on immune cell subsets. IL-18Rα was clearly present on Vδ2 T cells, whereas on other lymphocyte populations (CD4 and CD8 T cells, Vδ1 T cells) the surface abundance was much lower. While monocytes are major IL-18 producers, no IL-18Rα expression was observed. Only NK cells showed a comparably strong IL-18Rα abundance like Vδ2 T cells (Fig. 5A, left panel). Upon activation of PBMC with HMBPP, *M. tuberculosis,* or BCG, no qualitative change in the IL-18Rα expression on Vδ2 T cells was noted (Fig. 5A, right panel). The median fluorescence intensity, however, slightly increased upon *M. tuberculosis* or BCG stimulation (Fig. S4).

The maturation of IL-18 from pro-IL-18 is inflammasome-dependent. To further dissect the role of bacterial IL-18 induction, we therefore used the inflammasome inhibitor MCC950, which inhibits the maturation and release of IL-1β and IL-18. While MCC950 had almost no effect on the HMBPP-induced CD137 or IFN-γ upregulation by Vδ2 T cells, it clearly reduced IFN-γ production and, to a lesser extent, the CD137 upregulation upon *M. tuberculosis* and BCG activation (Figs. 5B and S5A). Compared to anti-IL-18, the effect of MCC950 on CD137 and IFN-γ upregulation on Vδ2 T cells was even more pronounced (Fig. S5A). A similar pattern as for IFN-γ was observed for TNF-α, which was strongly upregulated upon HMBPP activation, but to a lesser extent upon *M. tuberculosis* or BCG activation. Again, anti-IL-18 or MCC950 only reduced the bacterial induction (Fig. S5B). MCC950, in combination with the anti-BTN3A1 mAb 103.2, almost completely abrogated the CD137 and IFN-γ induction upon *M. tuberculosis* or BCG activation (Fig. 5B). Of note, NK cells within the PBMC also clearly reacted to the bacterial stimuli. CD69 and IFN-γ were induced in the presence of *M. tuberculosis* and BCG (Fig. S5C). Unexpectedly, we also noted some activation of NK cells by HMBPP, which resulted most likely from a bystander effect due to strong activation of γδ T cells within the PBMC. MCC950, as well as anti-IL-18 antibody, also diminished the bacteria-induced IFN-γ production by NK cells (Fig. S5C). Moreover, we also noted some CD4 T-cell activation by *M. tuberculosis* or BCG stimulation, quantified by the concurrent upregulation of CD154 and TNF-α production. Even though the response was moderate, it was also reduced by anti-IL-18 and MCC950 treatment (Fig. S5D).

## Discussion

We investigated the contribution of BTN2A1/3A, TCR signaling, and cytokines in the early activation of human Vδ2 T cells by Vδ2-selective pAg and various bacteria. The rapid upregulation of CD154 and CD137 is an established method to identify antigen-specific CD4 T cells and Treg for subsequent specificity and TCR repertoire analysis (17–22). Similarly, γδ T cells have been shown to upregulate CD137 upon stimulation with aminobisphosphonates and pAg (23, 33, 34). Such an assay was also applied to demonstrate the presence of allergen-specific γδ T cells in allergic individuals (35). Using the upregulation of CD137, CD154, and intracellular IFN-γ as a read-out for rapid activation of Vδ2 T cells, we observed striking differences in the requirement for BTN and TCR signaling between pAg and bacterial stimulation.

It is well known that various bacteria stimulate human Vδ2 T cells. This activation is generally attributed to the strong stimulatory capacity of bacterial pAg, which are intermediates of the nonmevalonate pathway of isoprenoid synthesis. Early studies have shown that lysates of *M. tuberculosis* specifically activate Vγ9Vδ2 T cells (24). Subsequently, nonproteinaceous pyrophosphates were isolated from *M. tuberculosis* and identified as the Vδ2 T-cell activating molecules (25). In the following, the usage of the nonmevalonate pathway of isoprenoid synthesis in bacterial species was found to determine their ability to induce γδ T-cell activation (1). γδ T-cell reactivity was also reported in the early stage of infections with *E. coli* and *S. aureus*, which was shown to be due to the accumulation of endogenous nonmevalonate metabolites (36). We confirmed that pAg play a role in bacteria-induced Vδ2 T-cell activation as it was partially reduced (especially for *M. tuberculosis*) upon BTN3 blockade. In contrast, however, the activation by pAg BrHPP and Zol was completely abrogated in the presence of anti-BTN2A1 or anti-BTN3A antibodies. Therefore, other factors apart from pAg-stimulation must be involved in the bacteria-induced CD137 upregulation in Vδ2 T cells. This notion is in line with previous observations of a pAg-independent Vδ2 T-cell activation that complements pAg-dependent activation. Even though the activation (based on upregulation of CD69, TNF-α and IFN-γ) of Vγ9 T cells within peritoneal leukocytes by extracts of *E. coli*, *K. pneumoniae*, *P. aeruginosa*, and *C. striatum* was blocked by anti-BTN3A mAb 103.2, the inhibition was not complete (37). Furthermore, HMBPP-nonproducing *S. aureus* and the HMBPP-deficient *L. monocytogenes* mutant still induced in vitro Vγ9Vδ2 T-cell activation and some expansion within the PBMC (37, 38). Given the intricate interaction of BTN2A1/BTN3A and the γδ TCR (primarily Vγ9) (7), the differential blocking activity of anti-BTN3A antibody points to differential involvement of TCR signaling in pAg *versus* bacterial stimulation. Therefore, we also analyzed the expression of Nur77 and the effect of TCR-blocking strategies to interrogate the role of TCR signaling in the short-term activation of Vδ2 T cells. Nur77 is a specific indicator of TCR signaling in human T cells (26). In line, Nur77 was readily induced in Vδ2 T cells upon activation with the pAg BrHPP and Zol but only very weakly upon bacterial activation. In further support of TCR-independent activation of Vδ2 T cells by bacteria, we observed that the Fab fragment of anti-Vγ9 mAb 7A5 (39) and the protein tyrosine kinase/LCK inhibitor Emodin, which inhibits downstream TCR signaling (27), had only minimal effects on CD137 upregulation by bacteria, while both reagents strongly reduced activation of Vδ2 T cells by BrHPP and Zol. Taken together, our results clearly identify a BTN/TCR signaling-independent component of the rapid activation of Vδ2 T cells in response to various bacteria.

In our study, we monitored the activation of Vδ2 T cells within PBMC. In fact, upregulation of CD137 was also observed in isolated γδ T cells in response to pAg but not in response to bacteria, suggesting that accessory cells (most likely monocytes) were required. Bacterial cell wall constituents might co-stimulate Vδ2 T-cell activation *via* innate receptors like Toll-like receptors (TLR). Vδ2 T cells have been demonstrated to express functional TLR (40). But even though γδ T cells can react to bacterial TLR ligands, such ligands have only a co-stimulatory role and alone are not sufficient to fully activate Vδ2 T cells, as has been shown for TLR2 stimulation (41–43). Other factors known to modulate T-cell activation independently of TCR stimulation are cytokines. Antigen-specific activation induces rapid upregulation of CD137 and CD154 on human Treg and Tcon, respectively (17, 18, 20–22). However, there are also several reports that CD137 and CD154 can be upregulated by cytokines, in particular by IL-15. For human CD4 T cells, it was demonstrated that the CD154 surface expression can be prolonged by IL-15. Moreover, it was demonstrated, that STAT5, which is induced by IL-15, binds to the CD154 gene-promotor and that STAT5 knock-down leads to a reduction in CD154 surface expression (29). Furthermore, CD137 upregulation was also demonstrated for murine CD8 memory T cells in response to IL-15 treatment (28). We found that the exogenous supply of IL-15 or IL-18 significantly enhanced the upregulation of CD137 on Vδ2 T cells, not only in response to BrHPP and Zol but also to bacterial stimulation. When we used neutralizing antibodies against a range of cytokines to block their possible endogenous production in our short-term activation assays, we observed that only IL-18 (and not endogenous IL-15) played an important role in the upregulation of CD137 on bacteria-activated Vδ2 T cells. Remarkably, there was an additive inhibitory effect of Vδ2 T-cell activation by bacteria in the presence of anti-BTN3A plus anti-IL-18 antibodies (Fig. 4B, C). Our results thus point to an important role for endogenous IL-18 in the BTN/TCR-independent component of Vδ2 T-cell activation by bacteria, notably *M. tuberculosis*. This was further confirmed in experiments where we blocked the inflammasome-dependent maturation of IL-18 with the inflammasome inhibitor MCC950. While MCC950 alone inhibited bacteria-induced Vδ2 T-cell activation to a similar extent as did anti-IL-18 antibody, the combined application of MCC950 plus anti-BTN3A antibody 103.2 almost completely abrogated bacterial Vδ2 T-cell activation. We conclude that the inflammasome activation and associated IL-18 production is a key factor in the BTN/TCR-independent Vδ2 T-cell activation by bacteria. Importantly, this applies not only to the activation with heat-killed bacteria but also to the stimulation with live bacteria, as shown here for BCG. Since we analyzed Vδ2 T-cell activation within total PBMC, the inhibition of the inflammasome most likely affected the monocyte population, which are the key producers of IL-18 within microbe-activated PBMC (44). However, the role of this inflammasome-dependent pathway in other accessory cells, such as dendritic cells or endothelial cells, which might also produce IL-18 upon infection and then co-stimulate Vδ2 T cells (45), remains to be addressed in further studies.

Such a co-stimulatory effect of IL-18 on the CD137 or CD154 induction has not been described before. However, this stress-related member of the IL-1 family is well known to be involved in the regulation of human Vδ2 T-cell activation, which is facilitated by the expression of the IL-18Rα chain on a substantial proportion of peripheral blood Vδ2 T cells (46, 47). In line, we observed IL-18Rα expression on Vδ2 T cells and NK cells, but much lower abundance on αβ T cells and Vδ1 γδ T cells. Several studies have reported that IL-18 alone or in combination with other cytokines (e.g. IL-12) upregulate activation markers CD25 and CD69 on Vδ2 T cells and co-stimulate proliferative responses, usually in combination with stimuli like pAg, Zol, or agonistic anti-TCR antibody (30–32, 48). Moreover, IL-18 can also enhance the cytotoxic effector function of tumor-reactive Vδ2 T cells (33, 49). In their study, Gruenbacher et al. (31) found that high concentrations (100 ng/mL) of IL-18 alone can upregulate the activation-related marker CD25 on Vδ2 T cells. The concentration of IL-18 used in our study (10 ng/mL) did not induce CD137 or CD154 expression by itself. Furthermore, since the maximum concentrations of IL-18 detected in the supernatants of the bacteria-activated PBMC (<0.8 ng/mL; Fig. S3) were even lower than what we used in this study as a medium supplement, an activation only by bacteria-induced IL-18 can be excluded in our study.

Our study identifies contrasting key features of Vδ2 T-cell activation by pAg and bacteria, notably *M. tuberculosis*. In line with many reports, the pAg-dependent activation of Vδ2 T cells completely depends on BTN2A1/BTN3A and TCR signaling, and there is no requirement for endogenous cytokines, including IL-18. In striking contrast, the activation by *M. tuberculosis* but also BCG only partially relies on BTN molecules, but also integrates additional signals, particularly those of the bacteria-induced and inflammasome-dependent IL-18. However, it is important to emphasize that IL-18 (in the concentration range of our experimental in vitro setting) per se is insufficient to induce the activation of Vδ2 T cells. Therefore, we postulate that a second activating signal, independent of TCR/BTN-signaling, must be involved in the IL-18-dependent activation pathway of Vδ2 T cells. While the precise molecular pathways remain to be discovered, our study provides new insights into the mechanisms of Vδ2 T-cell activation by microbes. The demonstration of a BTN-independent (and thus pAg-independent) stimulatory activity of at least certain bacteria for human Vδ2 T cells, together with the essential role of IL-18 in this process, may help to better understand the role of γδ T cells in infectious diseases. γδ T cells contribute to anti-infective immunity by the production of pro-inflammatory cytokines, including IFN-γ and TNF-α and the cytotoxicity towards infected cells (50). Our results indicate that substantial γδ T-cell activation may also occur in an IL-18-dependent context in response to bacteria lacking prominent production of pAg HMBPP and thus independent of BTN molecules. This could be envisaged in gastrointestinal or pulmonary bacterial infections with bacterial species devoid of the nonmevalonate pathway of isoprenoid synthesis, e.g. some species of *Enterococcus*, *L. monocytogenes*, *Strep. pneumoniae, Legionella,* and others (51). Furthermore, γδ T cells are receiving growing interest as effector cells in the adoptive immunotherapy of malignancies and severe infections (50, 52–54). From a translational perspective, our results thus also indicate that it might be useful to include exogenous IL-18 for the activation and expansion of Vδ2 T cells following Zol or pAg stimulation for subsequent use in adoptive cell therapy, since IL-18 enhances CD137 and CD154 upregulation (shown here) and proliferation (30). Moreover, this pathway of inflammasome-dependent activation could be of more general importance and might contribute to the activation of cell types from the innate immunity, such as NK cells, as well as cell types from the adaptive immune system, such as CD4 or CD8 αβ T cells.

## Materials and methods

### Blood donors and cell isolation

Blood from adult healthy donors was obtained as leukocyte concentrate from the Department of Transfusion Medicine, University Hospital Schleswig-Holstein Campus Kiel. All blood donors provided informed consent. The study was approved by the Institutional Review Board of Kiel University Medical Faculty (D462/22). Ficoll-Hypaque density gradient centrifugation was used for PBMC isolation. For magnetic isolation, pan γδ T cells were obtained by negative magnetic isolation using the TCRγδT cell isolation Kit (Miltenyi Biotec, Bergisch-Gladbach, Germany) according to the manufacturer's protocol. Pan γδ T cells were used for experiments when at least a purity of >95% was achieved.

### Flow cytometry

For cell surface labeling, the following mAb were used: anti-CD3 (clone: OKT3, Biolegend, San Diego, CA, USA), anti-CD4 (clone: SK3, Thermo Fisher Scientific, Waltham, NJ, USA), anti-CD8 (clone: SK1, Thermo Fisher Scientific), anti-CD14 (clone: MOP9, BD Biosciences), anti-CD25 (clone: M-A251, Biolegend), anti-CD56 (clone: REA196, Miltenyi Biotec), anti-CD69 (clone: FN50, Biolegend), anti-CD137 (clone: 4B4-1), anti-CD218 (IL-18Rα, clone: H44, Miltenyi Biotec), anti-CD154 (clone: 5C8, Miltenyi Biotec), anti-Vδ1 (clone: REA173, Miltenyi Biotec), anti-Vδ2 (clone: B6, BD Biosciences, Franklin Lakes, NJ, USA), and anti-TCRγδ (clone: 11F2, Miltenyi Biotec). For intracellular labeling, 3 μM monensin (Calbiochem/EMD Millipore, Burlington, CA, USA) was added to the activated cells 4 h before fixation. Subsequently, cells were fixed and permeabilized using the Foxp3/Transcription Factor Staining Buffer Set (eBioscience/Thermo Fisher Scientific). For intracellular staining, we used anti-IFN-γ (clone: 4S.B3, BD Biosciences), anti-TNF-α (clone: MAb11, Biolegend), and anti-Nur77 (clone REA704, Miltenyi Biotec). For dead cell discrimination, Live/Dead NIR (Thermo Fisher Scientific) was used. FACS samples were analyzed on a FACS-Fortessa flow cytometer (BD Biosciences) using the BD FACSDiva software v.8 or an Aurora spectral analyzer (Cytek) using the SpectroFlo 3.3.0 software. The FlowJo software v.10 was used for further analysis. An overview of the gating strategies used in flow cytometry-based experiments is presented in Fig. S6.

### Short-term activation assay

To detect Vδ2 T-cell specific activation within the PMBC, 1.5 × 10^6^ PBMC freshly isolated from healthy donors were seeded in a 96-well flat-bottom plate in RPMI 1640 supplemented with 5% heat-inactivated human AB serum, 100 U/mL penicillin, and 100 mg/ml streptomycin (AppliChem, Darmstadt, Germany). Anti-CD40 mAb (clone: HB14, Miltenyi Biotec) was added at 1 µg/mL to prevent CD154 (CD40-L) internalization during the short-term stimulation assay. Furthermore, 1 µg/mL anti-CD28 (clone: CD28.2, Biolegend) and IL-2 (10 IU, Novartis, Basel, Switzerland) were added as activation supporting agents. For the phosphoantigen-dependent activation, Bromohydrin pyrophosphate (BrHPP; 300 nM, Innate Pharma, Marseille, France), (*E*)-4-Hydroxy-3-methyl-but-2-enyl pyrophosphate (HMBPP; 100 nM, Echelon Biosciences, Salt Lake City, UT, USA), Zoledronate (2.5 µM, Novartis, Basel, Switzerland), and the agonistic BTN3A mAb clone 20.1 (5 µg/mL, ImCheck Therapeutics, Marseille, France) were used. Moreover, various heat-killed bacteria or fungi species (1 × 10^8^ cells/mL, Invivogen, Toulouse, France) were used. BCG was obtained from Medac (Wedel, Germany). After 16 h (or indicated time point) of incubation (37 °C, 5% CO_2_), various populations (CD3, TCR-pan-γδ, TCR-Vδ2) and activation markers (CD25, CD69, CD137, and CD154) or intracellular markers (IFN-γ, TNF-α) were labeled with the specific mAb and measured by flow cytometry. The short-term activation assay was performed in the presence or absence of inhibitory anti-BTN3A (5 µg/mL, clone: 103.2) or anti-BTN2A1 (5 µg/mL, clone: 7.48) antibodies (kindly provided by ImCheck Therapeutics). Additionally, TCR-dependent activation was blocked using the Fab-fragment of anti-TCR-Vγ9 mAb clone 7A5 (39) at 5 µg/mL or the _p56_LCK-inhibitor Emodin (Sigma-Aldrich, St. Louis, MO, USA) at 40 µM concentration. For the neutralization of cytokines, anti-IL-1α (1 µg/mL, clone: 4414, R&D/Bio-techne, Minneapolis, MO, USA), anti-IL-1β (0.5 µg/mL, clone: 4H5, Invivogen), anti-IL-6 (5 µg/mL, clone: MQ2-13A5, Biolegend), anti-IL-12 (10 µg/mL, clone: C8.6, Miltenyi), anti-IL-15 (10 µg/mL, clone: MAB2471, R&D/Bio-techne) and anti-IL-18 (0.1 µg/mL, clone: 2C10, Invivogen) were used. The inflammasome function was blocked using MCC950 at 5 µM (Invivogen).

### Quantification of cytokines in cell culture supernatants

For the quantification of analytes in cell culture supernatants, the bead-based multiplex assay Human Cytokine Panel 2 (Biolegend) was used.

### Statistical analysis and software

For statistical analysis (the respective tests are indicated in the figure legends) and visualization, Microsoft Excel 2010 and GraphPad Prism 9.5.1 were used. Also, R/RStudio 2022.07.1 with the package ggplot2 was used for visualization.

## Supplementary Material

pgaf358_Supplementary_Data

## Data Availability

The data underlying this article are available in the article and in its online Supplementary material.
