# Supplementary material for: Butyrophilin 3A/2A1-independent activation of human Vγ9Vδ2 γδ T cells by bacteria
Source: PNAS Nexus. 2025 Nov 13;4(11):pgaf358. doi: 10.1093/pnasnexus/pgaf358 (PMC12644456; doi:10.1093/pnasnexus/pgaf358)
Supplement: pgaf358_Supplementary_Data [file pgaf358_supplementary_data.pdf]

Supplementary information for

Butyrophilin 3A/2A1-independent activation of human Vγ9Vδ2 $\gamma\delta$ T cells by bacteria

Daniel Gombert[1], Jara Simeonov[1], Katharina Klein[1#], Sophie Agaugué[3], Alexander Scheffold[1], Dieter Kabelitz[1]* and Christian Peters[1,2] *

[1]Institute of Immunology, and [2]Cytometry Core Facility, Christian-Albrechts-University and University Hospital Schleswig-Holstein Campus Kiel, 24105 Kiel, Germany

[3]ImCheck Therapeutics, Marseille, France

[#]Current affiliation: Institute for Tumor Genetics, Kiel, Germany

*Equally contributing senior and corresponding authors

Email: dietrich.kabelitz@uksh.de; christian.peters@uksh.de

This file includes:

Supplementary Figures S1 – S6

Supplemental Figure S1

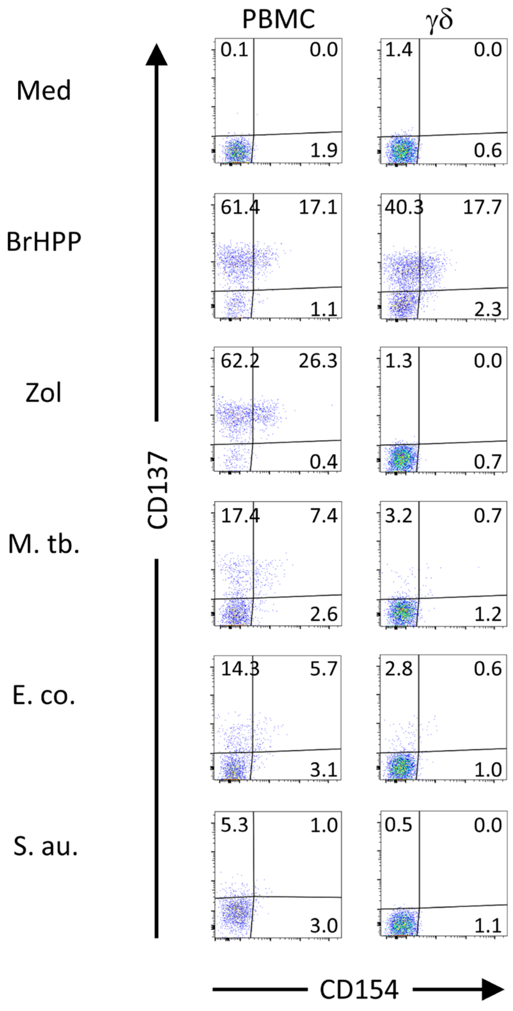

Activation of isolated γδ T cells compared to their activation within the PBMC. Untouched γδ T cells were negatively isolated from PBMC by magnetic cell sorting. PBMC and purified γδ T cells were stimulated for 16 h with BrHPP, Zoledronate [Zol], *M. tuberculosis* [M. tb.], *E. coli* [E. co.] and *S. aureus* [S. au.]. Expression of CD137 (Y-axis) and CD154 (X-axis) was analyzed on gated Vδ2 T cells. One representative out of three experiments is shown.

Supplemental Figure S2

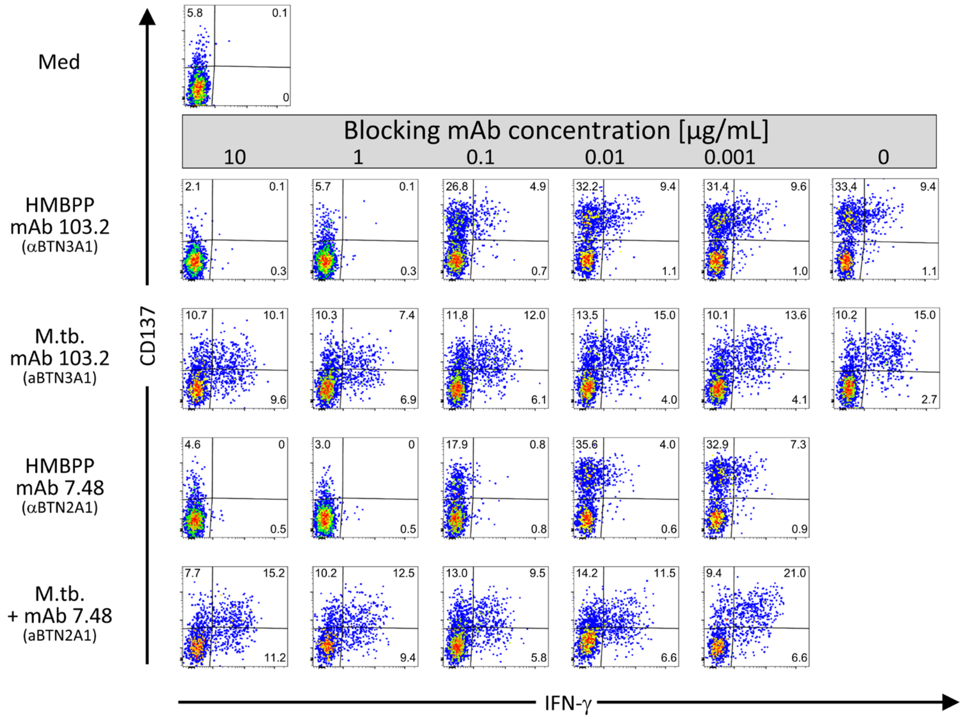

Titration of BTN-blocking antibodies. PBMC from healthy donors were stimulated with the phosphoantigen HMBPP, or heat-killed *M. tuberculosis* [M. tb.]. The surface mobilization of CD137 on Vδ2 T cells was measured by flow cytometry after 20 h. For the intracellular detection of IFN-γ, monensin was added 4 h before fixation. The dot plots of a representative experiment (out of three) show the effect of indicated concentrations (10 to 0.001 µg/mL) of inhibitory anti-BTN3A (clone: 103.2) and anti-BTN2A1 (clone: 7.48) antibodies on the CD137 and IFN-γ upregulation.

Supplemental Figure S3

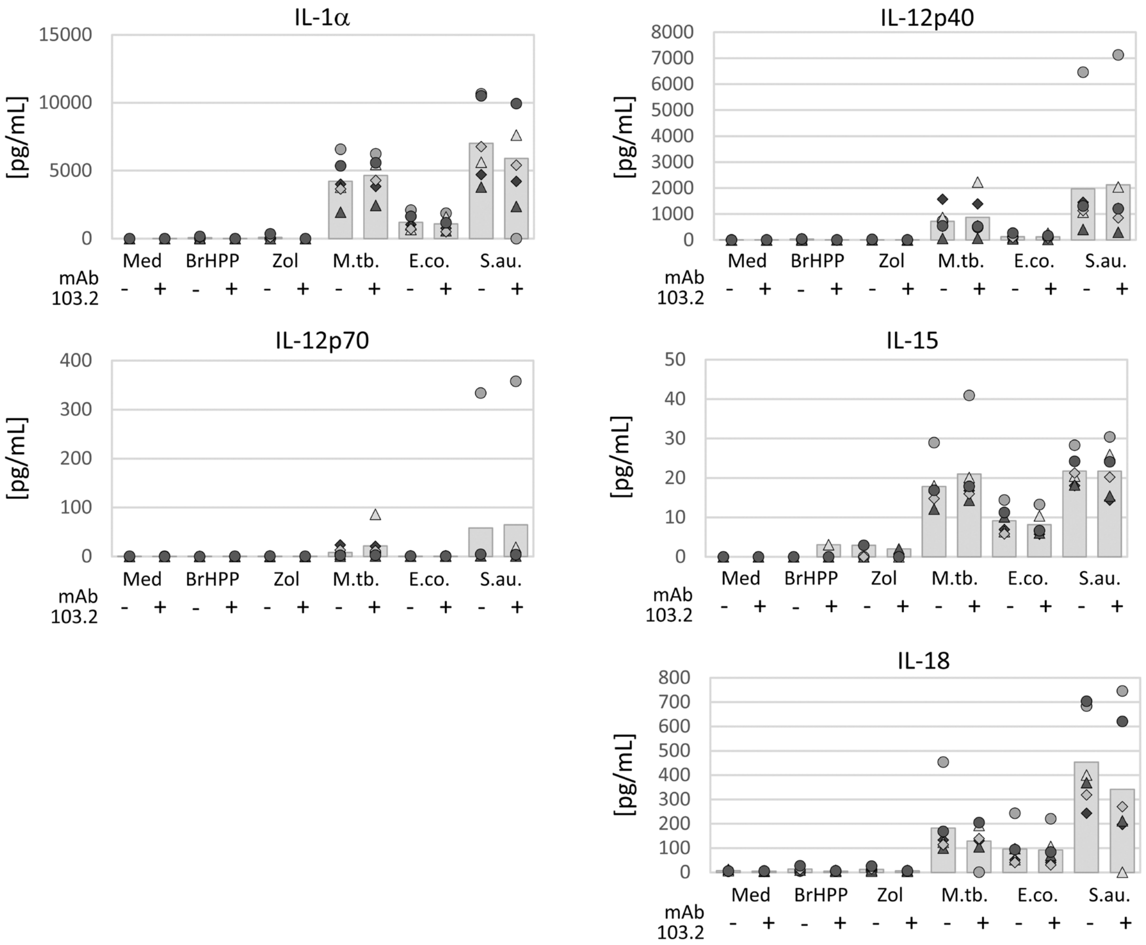

Cytokine levels in supernatants of bacteria-activated PBMC. Supernatants were collected from PBMC activated for 16 h with different Vδ2 T-cell selective stimuli (Zoledronate [Zol], BrHPP), or different heat-killed bacteria (*M. tuberculosis* [M. tb.], *E. coli* [E. co.], *S. aureus* [S. au.] in the absence or presence of inhibitory anti-BTN3A mAb 103.2. Different cytokines (IL-1α, IL-12p40, IL-12p70, IL-15, and IL-18) were quantified by a bead-based cytokine array (n=6).

Supplemental Figure S4

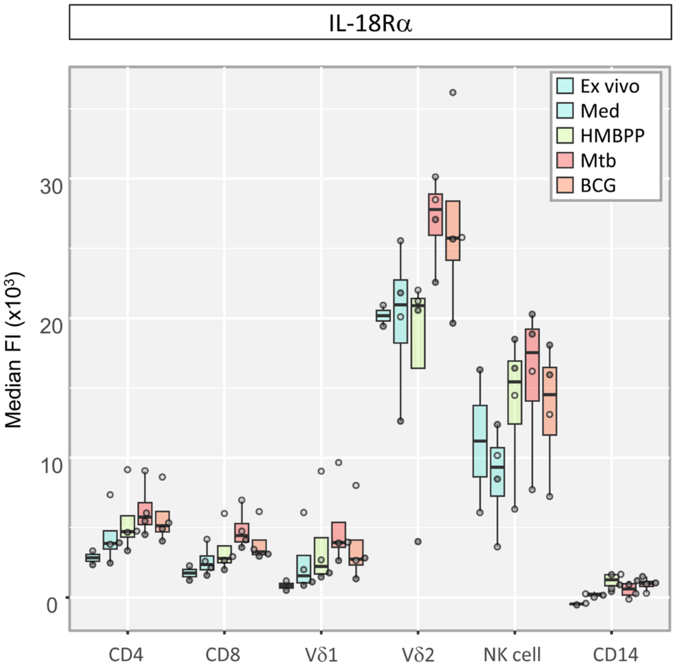

Effect of bacterial activation on IL-18Rα surface abundance. PBMC were stimulated for 20 h with HMBPP, *M. tuberculosis* [M. tb.], or Bacillus Calmette-Guérin [BCG], or remained unstimulated [Med, Medium]. The IL-18Rα (anti-CD218, clone: H44) surface abundance was measured by flow cytometry on different leukocyte populations (CD14+ Monocytes, CD14-CD3-CD56+ NK cells, Vδ1, Vδ2, pan-γδ, CD4 and CD8 T cells) within freshly isolated PBMC (ex vivo) or stimulated PBMC from four healthy donors.

Supplemental Figure S5

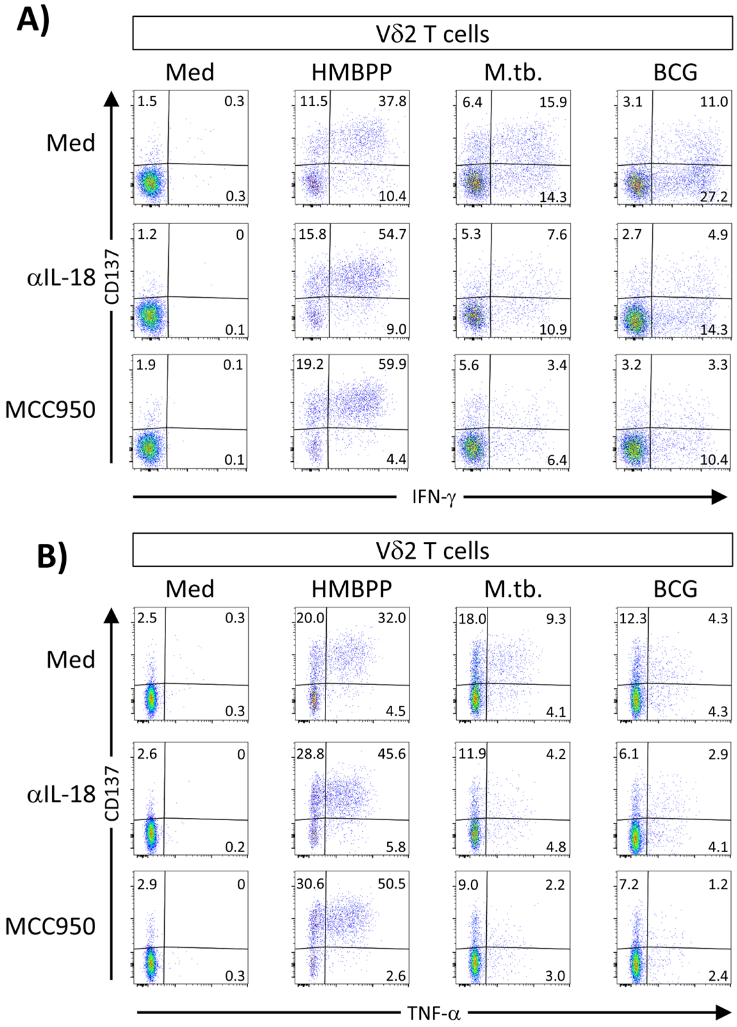

Supplemental Figure S5 continued

C)

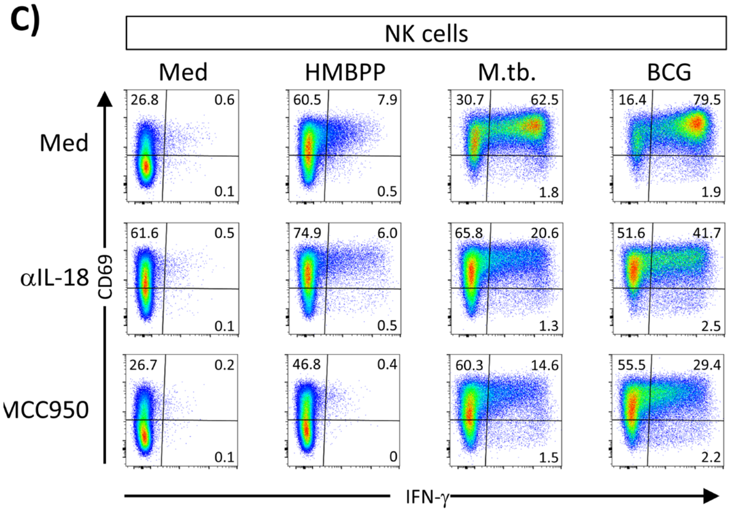

D)

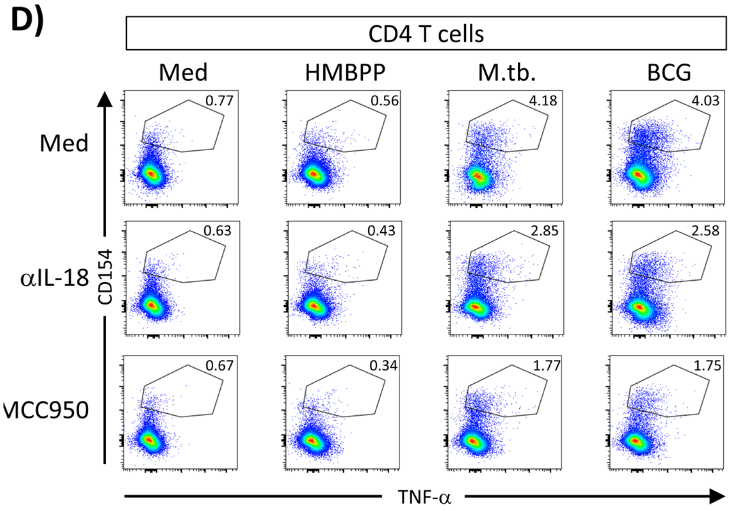

Inflammasome-dependent activation of Vδ2 T cells. PBMC were stimulated with the phosphoantigen HMBPP, heat-killed *M. tuberculosis* [M. tb.], live Bacillus Calmette-Guérin [BCG], or remained unstimulated [Med, Medium]. The surface mobilization of CD69 or CD137 was measured by flow cytometry after 20 h. For the intracellular detection of IFN-γ and TNF-α, monensin was added 4 h before fixation. The effect of anti-IL-18 and MCC950 on (A, B) Vδ2 T-cell, (C) NK-cell, or CD4 T-cell activation is shown in a representative experiment (out of four). Separate donors were analyzed in (A, B) and (C, D). The control panel showing the untreated [Med] Vδ2 T cells in (A, B) contains data already shown in Fig. 1D.

Supplemental Figure S6

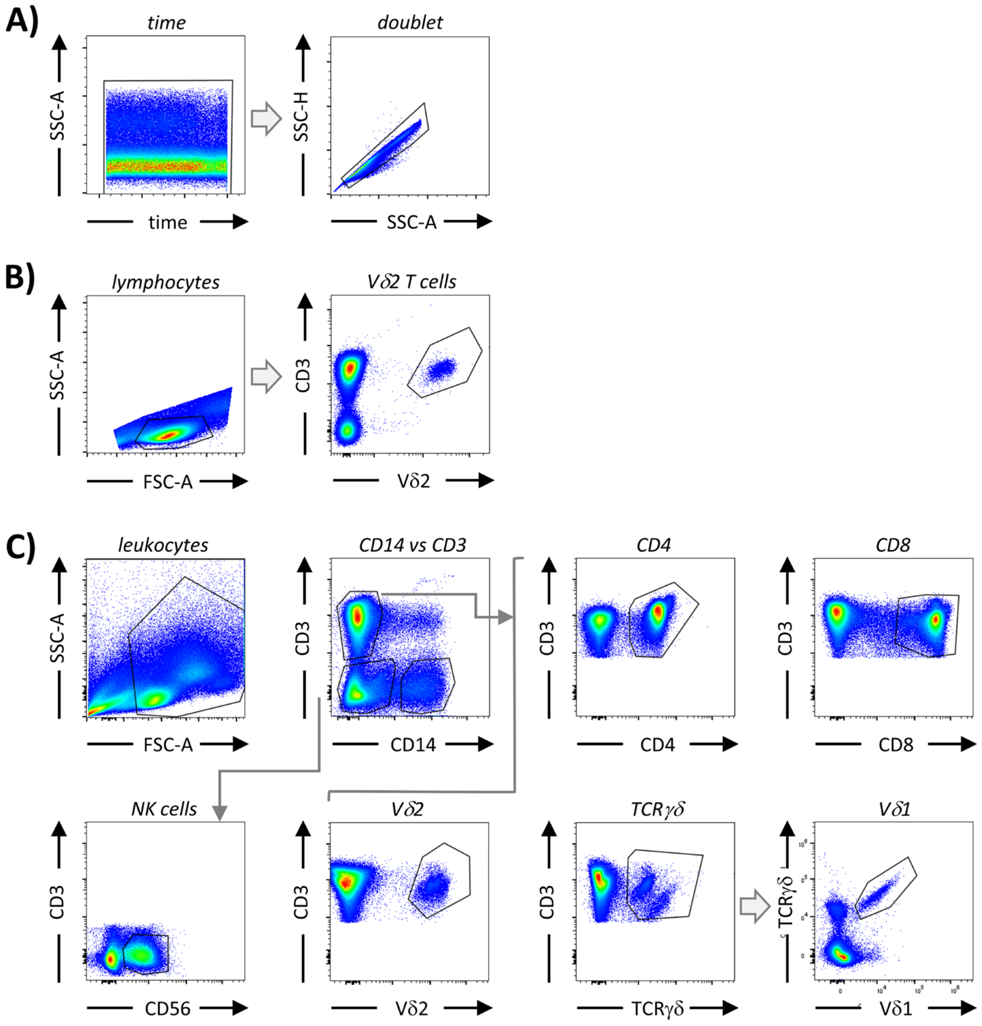

Gating strategy for flow cytometric analysis. (A) For all flow cytometric experiments, a pre-gating was used in which a time-gate, using the time and SSC-A parameter, followed by a doublet-gate using the SSC-H and SSC-A parameter. (B) For the experiments shown in Fig.1, 2, 3, 4, 5B and Suppl. Fig. 1, 2, in addition to the pre-gating, a lymphocyte-gate, using SSC-A and FSC-A parameter, was applied, followed by a Vδ2 T-cell gate on CD3+Vδ2+ cells. (C) For the experiment shown Fig. 5A, and Suppl. Fig. 4, 5 following the pre-gating, PBMC (using SSC-A and FSC-A parameter) and viable cells (using the FSC-A versus live/dead dye [L/D] channel) were gated. CD3+ cells were identified using the CD3 vs. CD14 parameter, followed by a gating on CD3+CD4+, CD3+CD8-, CD3+Vδ2+, CD3+TCRγδ+ cells. Subsequent to gating on TCRγδ, Vδ1+ cells were gated. CD14+ and CD3-CD14- cells were gated using the CD3 and CD14 parameter. From the double-negative population, the CD56+ population was gated.
